# Glaucoma glossary

**Published:** 2012

**Anterior chamber.** The part of the eye between the cornea and iris, filled with aqueous humour.

**Aqueous humour.** A clear fluid continually produced by the ciliary processes. It contributes to the maintenance of intraocular pressure. The fluid leaves the eye through the sieve-like trabecular meshwork and Schlemm's canal to reach deep veins in the sclera.

**Bleb.** A ‘blister’ of tissue overlying the site of glaucoma drainage surgery, from where aqueous escapes from the eye.

**Central vision.** The detailed vision in the centre of a person's gaze for which the macular area of the retina is used.

**Glaucoma.** A group of complex eye diseases characterised by optic nerve damage resulting in loss of vision with typical visual field defects, and, usually, with raised intraocular pressure.

**Intra-ocular pressure (IOP).** The pressure inside the eye that results from the combined production and drainage of aqueous humour, measured in millimetres of mercury (mmHg). Normal IOP ranges between 12 and 22 mmHg.

**Laser trabeculoplasty.** A surgical procedure to deliver a series of laser burns to the trabecular meshwork to improve the outflow of aqueous humour in open-angle glaucoma.

**Optic nerve.** The nerve tract that transmits visual information from the retina to the brain.

**Figure:**
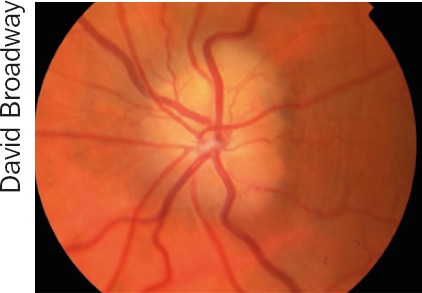


**Optic nerve head drusen.** Hyaline deposits, often calcified, in the region where the optic nerve enters the back of the eye. May be associated with visual field loss and may mask the presence of glaucoma.

**Perimetry.** A test that produces a map of the field of vision to plot visual field defects.

**Peripheral vision.** The top, sides, and bottom areas of vision. These may be the first areas of vision affected by glaucoma.

**Shunt.** An artificial drainage device surgically implanted in the eye to lower intraocular pressure.

**Trabecular meshwork.** A meshwork of connective tissue located at the angle of the anterior chamber of the eye and through which the aqueous humour drains.

**Trabeculectomy.** A well-established surgical treatment for glaucoma, in which a small, covered, drainage hole is created in the sclera to allow a controlled outflow of aqueous. An augmented trabeculectomy involves the local application to the trabeculectomy site of an agent that inhibits scarring.

